# Clinical trial methods for family medicine and primary care

**DOI:** 10.4102/phcfm.v17i2.5062

**Published:** 2025-07-25

**Authors:** Robert Mash, Bolatito B. Fatusin, Edith Madela-Mntla, Christopher Butler

**Affiliations:** 1 Division of Family Medicine and Primary Care, Faculty of Medicine and Health Sciences, Stellenbosch University, Cape Town, South Africa; 2 Federal Medical Centre, Abeokuta, Nigeria; 3 Department of Family Medicine and COPC Research Unit, University of Pretoria, Pretoria, South Africa; 4 Nuffield Department of Primary Care Health Sciences, University of Oxford, Oxford, United Kingdom

**Keywords:** clinical trials, randomised controlled trial, experimental studies, study design, adaptive platform design, methodology, methods, primary care

## Abstract

This article outlines the essential features of clinical trials for doctoral or early career researchers. The World Health Organization has recently emphasised the need for higher quality clinical trials, more trials from low- and middle-income countries, as well as primary care, more engagement with patients and communities and adoption of innovative trial designs. In sub-Saharan Africa, primary care researchers need to move beyond quasi-experimental and before-and-after designs to conduct randomised clinical trials. The article describes the key methodological requirements of a randomised controlled trial: the hypothesis, design, setting, recruitment, randomisation, sample size, intervention, assessment, results, interpretation and extrapolation. We also discuss the aspects of ethical and well-organised trials that respect study participants, engage with collaborative processes, have appropriate governance and transparent dissemination of results. Finally, we outline innovative designs such as step-wedge, clinical trial networks and adaptive platform designs.

## Introduction

Randomised controlled trials (RCTs) are considered the most robust research method for determining whether interventions are effective. They are a form of experimental study in which an intervention of any kind is tested. This could be a drug, a medical device, a natural product, a preventative manoeuvre, a behavioural therapy or a change to the service delivery model. The intervention is either delivered or not delivered according to a process of chance, or randomisation, so known and unknown differences between those exposed to the intervention and those who are not are balanced. The intention is that the only major difference between the groups is the experimentally manipulated exposure to the intervention. Despite the importance of such experimental studies, they make up only 2% of all original research published in the *African Journal of Primary Health Care and Family Medicine*.^1^ Even in developed countries, there are far too few trials in and on primary care. A UK Government-commissioned report on commercial trials in the UK highlighted that only 10% of clinical research activity involves primary care and only 4% of practices participate in commercial trials.^2^

Clinical trials are often too resource and time-intensive for master-level students to tackle, but as this series is aimed at doctoral students and early career researchers, we hope that clinical trial methods will be increasingly adopted. This article aims to describe the key methodological issues and an overview of some of the new and emerging methodologies that are relevant to family medicine and primary care.

There are many reasons why clinical trials are less common in low- and middle-income countries (LMICs) as well as in family medicine and primary care.^3^ Such trials have traditionally been conducted in tertiary hospitals on patients who may not be typical of primary care, and yet the results are extrapolated. In LMICs, there is a dearth of electronic data, a lack of research expertise, insufficient national research funding, complex regulatory frameworks and insufficiently trained ethics review boards. These barriers are frequently multiplied for family medicine and primary care researchers, and primary care services are often under huge clinical pressure. In 2024, the World Health Organization published guidance to improve the quality and usefulness of clinical trials,^4^ and in 2025, a global action plan (Box 1).^5^

BOX 1 World Health Organization global action plan.

Action 1: Strengthen local leadership and national support for sustained infrastructure and funding

Action 2: Enhance involvement and engagement with patients, communities and the public in clinical trial lifecycle

Action 3: Address barriers to clinical trials in under-represented populations

Action 4: Enable effective trials through the adoption of innovative designs and digital technologies

Action 5: Accelerate access to fit-for-purpose training packages for clinical trials

Action 6: Improve coordination and streamlining regulatory and ethics review

Action 7: Engage clinical practitioners to integrate clinical trials into health systems and practices

Action 8: Step up the use of trial registries to improve research transparency

Action 9: Expand international health research and clinical trial collaboration

*Source:* Adapted from World Health Organization. Global action plan for clinical trial ecosystem strengthening. Geneva: World Health Organization, 2025

## Experimental studies

Before focusing on RCTs, it is useful to spend a moment considering other experimental study designs. While these designs are less robust, they may be more feasible and still produce useful results.

Quasi-experimental studies share many of the design features of an RCT, but the participants are not allocated randomly to groups. Random selection of patients in RCTs is intended to ensure that the comparison groups are similar in all their characteristics, both known and unknown, and so differ only in their exposure to the intervention. In health services research, however, it is not always possible to assign patients randomly, as such groups may already exist as natural experiments. When reporting on such studies, one must consider the likelihood that the results are because of different characteristics in the two groups and not simply exposure to the intervention.

Another design is the before-and-after study and the interrupted time series study, if several observations are included over time. This approach follows the same group of people from before to after an intervention. Such studies can be retrospective, where existing data are used to look back in time, and this approach can be relatively quick and low-cost. Prospective studies follow people forward in time and have the advantage of being able to collect data that are extra to routine data for the purposes of the study, called a hypothesis-driven cohort study. The advantage is that the data are often more fit for purpose and can be more complete, but these studies are more time-consuming and expensive. The Achilles heel of this approach is the absence of a control group and, again, any effects could be because of unmeasured changes or unknown interventions in the group and not because of the focus of the experiment. Again, one would need to consider the likelihood of such factors being responsible for any effects when reporting on the study.

These types of experimental studies are more difficult to publish, and the results are regarded with some scepticism because of the potential for bias and error. In an ideal world, researchers should attempt to design and conduct an RCT when answering questions on effectiveness.

## Randomised controlled trials

**FIGURE 1:**
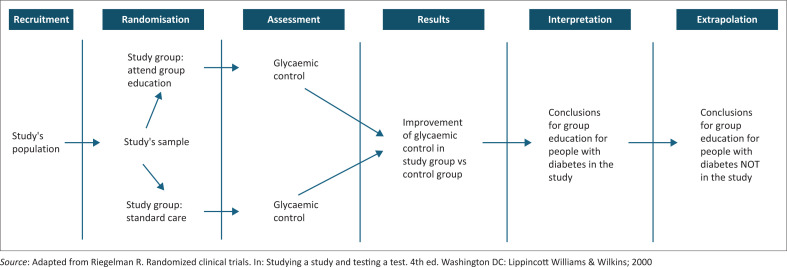
Key features of a randomised controlled trial.

The classic RCT is well described in many books and journal articles. The CONSORT statement was created to define the essential features of the methods and what should be reported.^6^ Figure 1 illustrates the key features of an RCT where the intervention is group diabetes education.^7^

### Aim and objectives

The aim and specific objectives of the study should be clearly stated, as well as the underlying hypothesis of the experiment, for example, that treatment or approach A is more effective than placebo or standard care.

The discourse on clinical trials usually focuses on medications, but primary care providers may also be interested in the effectiveness of behavioural interventions, diagnostic tests, health promotion and disease prevention or changes to service delivery and the model of care. Core functions such as access, utilisation, continuity, coordination, comprehensiveness and person-centeredness might be important outcomes. Health gains from answering such health services research questions can be considerable.

### Study design

In clinical pharmacology, when a new drug is being tested, trials are labelled as *phase I to phase III trials*:^7^

- Phase I trials administer the drug to humans for the first time, establishing a dosage regimen and identifying any obvious toxicities. They are not concerned with efficacy per se.
- Phase II trials may be small-scale RCTs or uncontrolled trials to establish ‘proof of principle of efficacy’ or whether a larger RCT should be conducted. They are not usually sufficiently powered to be able to demonstrate efficacy.
- Phase III trials are larger-scale RCTs that are designed to determine the efficacy of the new treatment.

In primary care, however, we often focus on the effectiveness of an intervention in conditions closer to the real world rather than efficacy trials. Trials that embrace real-world conditions and more diverse participants are called *pragmatic trials*. In pragmatic trials, we see if an intervention, which may be efficacious under ideal conditions, can also deliver the expected effects in the actual health services. There are many examples where effect sizes (the size of the effect on the intervention group compared to the control) achieved in efficacy trials, in tightly controlled circumstances and homogenous populations, are not replicated in trials done in real-world circumstances. Primary care clinicians are right to ask whether the participants in published trials are similar enough to the kinds of patients they see in their own practice; in other words, we must all ask the question, ‘how might the results of a trial be applicable to my patients, given the circumstances in which the trial was done, and the characteristics of the trial participants?’

In real-world conditions, it is sometimes necessary to allocate whole groups of participants linked in some way to an intervention rather than individuals. For example, in a trial looking at group empowerment of people with type 2 diabetes, it might be difficult to avoid contamination of the control group by the intervention group when they all share the same health centre. In this example, certain health centres were randomly allocated as controls and others as intervention sites. The trial then consisted of *clusters of participants* at each health centre who might also share certain similarities, and the effect of clustering must be taken into consideration when calculating the sample size and in analysing the results. Typically, in cluster randomised trials, increasing the number of clusters gives more power than increasing the number of participants in each cluster. This is because participants in each cluster are likely to be similar to each other in important ways, so cannot be seen as truly independent observations, as in individually randomised trials.

Typically, the intervention and control groups are studied in *parallel* at the same time. In primary care, it may also make sense to compare a new treatment to the current standard of care rather than a placebo. This will help decision-making on whether the intervention really adds value to existing primary care practice. In *factorial designs,* the trial may compare several interventions at the same time and have more than two groups.

Before conducting an RCT, it is recommended that the trial be registered in a trial registry, and in many cases, the full protocol is published. This makes it easier for the work to be found by others and ensures that the trial design is not altered at a later date to fit the data or results. When the RCT is completed, the results can also be included in the register, making the new evidence easily accessible.

### Setting

As with all studies, it is important to describe the setting and location in which the RCT took place. For example, the reader needs to know if this was a tertiary hospital or a primary care setting.

### Recruitment

People are recruited according to strict *inclusion and exclusion criteria*. They are not randomly selected for inclusion as in a survey. In efficacy trials, these criteria may exclude many people, for example, the very young, the very old, people with target organ damage, pregnant mothers or people with comorbidities. There may also be unintended bias because of the location of the trial in terms of socio-economic status or ethnicity. Having a very homogenous group makes it easier to test the hypothesis but makes the results more difficult to extrapolate to clinical practice. The dates and period for recruitment should also be stated.

### Randomisation

A cardinal feature of an RCT is that people are *randomly allocated* to an intervention or a control group. Usually, allocation is equal between groups, but in some cases, an *allocation ratio* may be used so that the groups differ in size.

The method used to generate the *random allocation sequence* should be described. In addition, the mechanism for implementing this in practice and *concealing the allocation* until it is made should also be described. Who generates the sequence, who recruits participants and who assigns participants should be stated. This is important to ensure that researchers cannot bias the allocation of participants to particular groups.

Randomisation is seen as important because it ensures that people in the control and intervention groups are the same apart from exposure to the intervention. Having a *control group* is important so that the two groups are exposed to all the same factors, apart from the intervention. This ensures that, as far as possible, any effects are because of the intervention and not differences in the people or to other factors that they are exposed to.

### Sample size

The sample size calculation should be described with all the assumptions made. The sample size should be calculated based on the characteristics of the primary outcome measure. The effect size can be determined by estimating the proportion of the control group who are expected to experience the outcome and the change expected in the intervention group that would be clinically meaningful.

**FIGURE 2:**
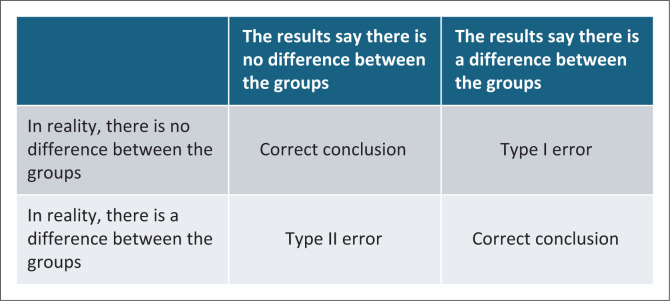
Types of error in a randomised controlled trial.

The amount of type 1 and type 2 errors allowed should be defined (Figure 2).^7^ A type-I error is when the study concludes that there is a difference between the groups, when, in reality, there is not. This is the most dangerous error as it may imply an intervention is effective when it is not. Typically, researchers allow for a 5% type-I error.

A type-II error concludes there is no difference between the groups, when, in reality, there is. This is considered less dangerous, although effective interventions may be missed. Researchers often allow for a 10% error and may go as high as 20%. The reciprocal of this error is called the power of the study (i.e. 90% power, for a 10% error rate).

### Intervention

The intervention given to each group should be explained in detail, including how and when it was administered. This includes what happened to people in the control group.

### Assessment

*Primary and secondary outcome measures* should be pre-defined to measure the objectives and test the hypothesis. Any changes to these outcomes after the RCT started should be stated and justified. Examinations should be defined in terms of standard operating procedures (e.g. weight or hip circumference). Laboratory or point-of-care tests should be specified as well as their accuracy. Questionnaires should be described along with their validity. How and when these assessments are made should be clearly described.

*Blinding* refers to the processes used to ensure that participants, researchers, and care providers are unaware of which group the person is allocated to. If researchers are aware of the participant’s group, this may influence the results of the assessments made. If outcomes are more subjective, then the participant’s knowledge of which group they are in could influence their behaviour or response. If care providers are aware of the participant’s group, it could influence their clinical behaviour. Blinding, therefore, is a way of minimising these sources of bias. However, it is not always possible to ensure blinding. For example, participants will know if they attended group education sessions or not. Even when the design intends to blind people, it may be necessary to check if this was successful. For example, even if intervention and control tablets look the same, the side effects may enable participants to guess which group they are in.

**FIGURE 3:**
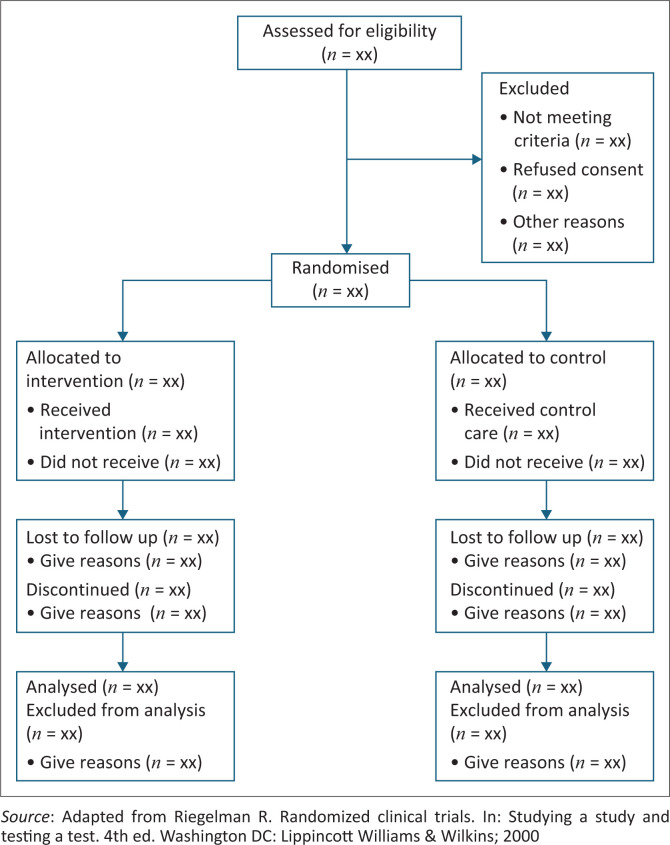
Flow diagram of a randomised controlled trial.

A *flow diagram* (Figure 3) should be planned to show how many people were recruited and allocated to each group, how many received the intended intervention and how many were included in the analysis of the primary outcome.^7^ Losses and exclusions should be explained. All the participants must be accounted for on follow-up and by the end of the trial.

When there is substantial *loss to follow up,* this may limit the validity of the results. For example, if a certain type of participant was lost because of more advanced disease or side effects, the remaining participants may have an artificially better outcome. One could assume that all those lost to follow-up had a poor outcome, but this may result in ambivalent results. Sometimes, missing data can be statistically imputed, and if this is done, then the method should be described.

Likewise, some participants may remain in the trial but deviate from the set protocol. An *intention-to-treat analysis* is seen as a standard whereby the participants allocated to a group are included in the main analysis whether they received the expected treatment or intervention or not. However, a secondary analysis is often done in which those participants who are sufficiently exposed to the interventions are analysed. If there are many *protocol deviations*, then the study may start to evaluate effectiveness more than efficacy (i.e. whether prescribing a treatment has a better outcome vs whether receiving a treatment has a better outcome).

A biostatistician is usually required to support or conduct the analysis. The protocol or report should describe the *statistical tests* used to compare groups for the primary and secondary outcomes. It should also explain any additional analyses, such as subgroup analyses or adjusted analyses.

## Results

In the results section, the baseline demographic and clinical characteristics of each group are presented so the reader can see if they were similar at the start of the trial. Results for each primary and secondary outcome should be presented, along with the estimated effect size. Numerical data can be presented with 95% confidence intervals, while categorical binary outcomes can be presented with both absolute and relative effect sizes. The frequency of harms or adverse effects should also be reported.

### Interpretation

Researchers may be tempted to highlight statistically significant findings and draw overly positive conclusions, even if the effect size is not clinically significant. We all like to report positive results. Studies with negative results also have a lower chance of being published. The relative effect size can be particularly misleading if the absolute effect is not also considered.

For example, one study of statins to prevent ischaemic heart disease found that after five years, 7.9% of controls had an event (fatal or non-fatal myocardial infarction), compared to 5.5% of those taking the statin.^8^ The relative risk reduction was calculated as 30%, while the absolute risk reduction was only 2.4%. Doctors and patients are much more likely to be impressed by a 30% risk reduction than a reduction in risk of only 2.4%. One can also calculate the number needed to treat to prevent one event (1/absolute risk reduction), which in this case would be 42 people for five years.

The frequency of harm or adverse events is usually small in the sample sizes recruited for clinical trials, and differences are not often statistically significant. However, an RCT is not designed to detect adverse events, and such events may still be clinically significant. Likewise, rare but serious adverse events may not be detected at all. For example, penicillin anaphylaxis occurs one time in every 10,000 treatments. According to the rule of three, to have a 95% probability of detecting one case, you would need to treat 30,000 people.^7^ Very few RCTs would have numbers on this scale. This is why ongoing surveillance of new drugs and reporting is essential in clinical practice.

### Extrapolation

When reading a report of an RCT, it is important to look at the inclusion and exclusion criteria and the reported characteristics of those in the trial. Are the participants similar to your own patients? If not, you may need to be wary of extrapolating the results. In efficacy trials, the participants may be more adherent to treatment than typical patients. Participants from tertiary hospitals may have more advanced disease than those in primary care. Care providers in the trial may have more expertise or better equipment than those in family practice. Practitioners tend to extrapolate beyond the evidence base to meet the needs of practice, but should do so with awareness and caution.

## Ensuring ethical and well-organised clinical trials

Trials should be conducted according to the principles of the Declaration of Helsinki, which has been recently updated.^9^

### Respect for the study participants

Information on a clinical trial should be co-designed with patients, the public or local community to ensure it is thorough, culturally sensitive, understandable and in the right languages.^4^ While all important information should be given, the researcher should avoid excessive detail that overwhelms the participant and may obfuscate more than explain. Full informed consent should be obtained as guided by a research ethics committee. Participants should be free to withdraw from the trial at any time. However, understanding the person’s concerns may allow them to continue by changing the terms of their consent rather than withdrawing completely and losing all their data. The safety of participants should be paramount and continuously reviewed, typically by an independent data monitoring committee, which can also unblind participants should this be necessary to their medical management.

### Collaborative processes

Trials often involve a collaboration between researchers, health services, commercial businesses or funders, and participants from the local community.^4^ Successful trials build trust through open communication and collaboration. A sharing of ideas and perspectives usually helps to strengthen the design, appropriateness and acceptability of the trial.

This is particularly important with potential participants or community members who can contribute to the design, implementation and interpretation of results. Of course, such community participation also ensures that local needs, diverse groups and perceptions are understood and addressed. Usually, a committee or structure is established to ensure community engagement and participation.

In global health practices, it is common for funding and expertise to come from high-income countries, while the participants and implementers are in LMICs. Collaboration should ensure that relationships are equitable, fair, and respectful.

### Governance of trials

Trials need to be well managed with sufficient oversight to ensure that they produce useful information in an efficient and ethical manner.^4^ A trial steering committee should monitor progress and provide advice to ensure scientific and ethical integrity. The committee should have independence from sponsors or those with vested interests. Excessive bureaucracy and governance processes should, however, be avoided. Research ethics committees may also monitor, audit and inspect trials to an extent that is proportional to the risks involved.

### Transparency and dissemination of results

The trial protocol, statistical analysis and results should be publicly available in a clinical trial registry.^4^ Thought should be given to data sharing when planning the trial and obtaining informed consent. The results, whether positive or negative, should also be published in a scientific peer-reviewed journal, ideally one that is open access. Plans should be made on how to best disseminate the results to all stakeholders from policy makers to the participants and public.

## Future directions and innovative designs for primary care

### Step-wedge designs

When a complex intervention is likely to remain as part of the care provided, then a stepped wedge design may be appropriate. In this design, clusters, for example, clinics, can start a new way of doing things in a staggered fashion according to randomisation. While one clinic or cluster is waiting to be initiated in the new way of doing things, it provides control information against which information from the participants in a cluster that is now delivering the new complex intervention can be compared. In time, the control cluster is trained and initiated in the novel intervention, and results can be compared to other clusters that have or are providing control information as they are not yet initiated.

### Practice-based clinical trial networks

One of the weaknesses of the traditional approach to conducting a single RCT is that researchers set up the research team and the locations and then dismantle them at the end of the trial. Often, the expertise and infrastructure are lost, and the whole process is very time-consuming and inefficient, as you start each time from scratch.

In primary care, there is an emergence of practice-based research networks.^10^ These are networks of practising primary care providers who are interested in participating in research. They may not have the expertise to design the study, write the proposal or analyse the data, but can identify important research questions from clinical practice and collect the data. In a large network, each primary care provider may only need to collect a relatively small amount of data, which makes it feasible. At the same time, the network may cover a wide area, such as a district or province, making the findings of more scientific value. Academic primary care researchers at a university or research institute can support the network with methodology, analysis and scientific writing. It can be important to ensure that research questions are prioritised by the network members and avoid external entities using the network for their own purposes. Authorship and recognition should be shared appropriately in the network. Such a network can participate in multiple consecutive research projects and become an engine for generating relevant primary care research.

It is a short step away from this to establishing a clinical trial network in primary care. If the focus of the network becomes clinical trials, then it will be important to train the members in the key principles and invest in sufficient space, expertise and information technology. Clinical trials can then be implemented within an established platform or network without the need to constantly develop a new research team and locations.

Such networks may value a more democratic and participatory approach between the clinicians and researchers. Increasingly, the participants’ voices are also included to co-design and develop the intervention in a way that is acceptable and feasible for them and to ensure that they are respected in the process. The social and scientific value of the work should be shared. Funders are also increasingly requiring community engagement and involvement in the whole research process.

### Adaptive platform designs

This type of clinical trial was used very effectively during coronavirus disease 2019 (COVID-19) to test the effectiveness of multiple treatments. An adaptive design means that the design of the trial can be modified after it starts. This thinking deviates from the traditional approach that a trial protocol is pre-determined and cannot be changed. The design can be adapted to address new questions or even modify the statistical procedures, without a loss of rigour. The idea of a platform implies that the trial infrastructure can be used to test multiple interventions during the trial. Interventions may be discarded, combined or added as the investigation proceeds.

For example, in one project looking to improve access to eye care in LMICs, the initial research generated several potential interventions.^11^ As people were screened and identified in the community, they were referred to the local eye clinic and randomly allocated to the standard of care or to receive an intervention (e.g. enhanced verbal counselling at the point of referral). The primary outcome was accessing the eye clinic. Interventions were continued until a predetermined probability of being effective was reached.^12^ Thus, the sample size was determined by reaching this probability or not and was not predetermined. If the intervention failed to reach this probability, then it could be discarded or boosted by a new intervention (e.g. enhanced SMS reminder on the day of referral). The trial could then continue with this new intervention. This approach is also more efficient as the researcher is not required to reach a certain sample size and can move on if a smaller sample size demonstrates effectiveness.

Examples of platform adaptive trials from the COVID-19 pandemic include the UK Urgent Public Health PRINCIPLE trial of repurposed medicine to treat acute COVID-19 that produced the world’s first, robust RCT evidence from primary care that azithromycin and doxycycline did not work for COVID-19.^13^ Unnecessary use of these drugs drives antimicrobial resistance, wastes resources and puts patients at unnecessary risk of side effects. Findings from the trial supported a Clinical Alert from the Chief Medical Officers to all National Health Service (NHS) clinicians that advised against the use of these drugs for COVID-19. PRINCIPLE also found that the widely promoted anti-inflammatory drug, colchicine, was ineffective for COVID-19, and that ivermectin and favipiravir did not result in clinically meaningful improvements in the short or longer term. It showed that inhaled budesonide (a cheap, safe inhaled steroid used for asthma) treatment helped largely unvaccinated people with COVID-19 recover faster and had a high probability of reducing the need for hospital care, and was permitted to be used for a time in the NHS. Findings on inhaled budesonide were taken up into guidelines in several countries, including India, Switzerland, Brazil, the Netherlands and Canada.

The PANORAMIC trial found that the novel antiviral molnupiravir did not reduce the need for hospital admission in vaccinated people with COVID-19 at higher risk, but helped people recover quicker; once well, it helped people stay well more often; reduced consulting in primary care; and reduced secretion of the SARS-CoV-2 virus.^14^ However, by day 14, similar amounts of virus were recovered from those who used the drug and those who did not, suggesting that the five-day treatment course is too short. Benefits for long COVID were modest. Molnupiravir is not cost-effective at its current price point. These findings supported National Institute for Health and Care Excellence (NICE) and World Health organization (WHO) Living Guidelines for the treatment of COVID-19, and guidelines in most countries worldwide, and continue to inform policy, procurement, research methods, and antiviral stewardship and care decisions worldwide.

It can take about four years for a traditional two-arm trial to produce results. If we had followed the model of sequential two-arm trials rather than using an adaptive trial design, generating the evidence achieved in PRINCIPLE and PANORAMIC would have taken around 30 years rather than the actual five years!

As primary care researchers reflect on the appropriate design of clinical trials for their context, there is also interest in new ways to measure outcomes. Traditionally, outcomes have required a standardised examination, administered tool or investigation for biomarkers. It may, however, be acceptable to use patient-reported health outcomes (e.g. mobility), for patients to self-sample at home (e.g. nasal swab) or to use routinely collected data. In LMICs, of course, some of these options may be difficult as systems are not electronic and engaging people at home is not easy.

## Conclusion

Doctoral and early career researchers should not shy away from the possibility of a clinical trial. The WHO is advocating not only for better-quality trials with less research wastage but more trials in primary care and LMICs. Trials can not only test the effectiveness of new medications but also consider behavioural and service delivery interventions in the primary care context. This article outlines the key methodological ingredients in a high-quality trial and points the way towards newer study designs that may be particularly appropriate for primary care researchers and practice-based networks.
